# Deep Learning-Based Detection of Glottis Segmentation Failures

**DOI:** 10.3390/bioengineering11050443

**Published:** 2024-04-30

**Authors:** Armin A. Dadras, Philipp Aichinger

**Affiliations:** Speech and Hearing Science Lab, Division of Phoniatrics-Logopedics, Department of Otorhinolaryngology, Medical University of Vienna, Währinger Gürtel 18-20, 1090 Vienna, Austria; armin.dadras@meduniwien.ac.at

**Keywords:** failure detection, deep learning, computer vision, glottis segmentation, semantic segmentation, high-speed videolaryngoscopy

## Abstract

Medical image segmentation is crucial for clinical applications, but challenges persist due to noise and variability. In particular, accurate glottis segmentation from high-speed videos is vital for voice research and diagnostics. Manual searching for failed segmentations is labor-intensive, prompting interest in automated methods. This paper proposes the first deep learning approach for detecting faulty glottis segmentations. For this purpose, faulty segmentations are generated by applying both a poorly performing neural network and perturbation procedures to three public datasets. Heavy data augmentations are added to the input until the neural network’s performance decreases to the desired mean intersection over union (IoU). Likewise, the perturbation procedure involves a series of image transformations to the original ground truth segmentations in a randomized manner. These data are then used to train a ResNet18 neural network with custom loss functions to predict the IoU scores of faulty segmentations. This value is then thresholded with a fixed IoU of 0.6 for classification, thereby achieving 88.27% classification accuracy with 91.54% specificity. Experimental results demonstrate the effectiveness of the presented approach. Contributions include: (i) a knowledge-driven perturbation procedure, (ii) a deep learning framework for scoring and detecting faulty glottis segmentations, and (iii) an evaluation of custom loss functions.

## 1. Introduction

Medical image segmentation plays a pivotal role in various clinical applications, aiding in the precise delineation and characterization of anatomical structures. However, despite advancements in segmentation algorithms, the automatic extraction of anatomical regions from medical images remains challenging due to factors such as noise, imaging artifacts, and anatomical variability. Consequently, inaccurate or failed segmentations can adversely impact downstream tasks, including disease diagnosis, treatment planning, and outcome prediction.

High-speed video (HSV) recordings offer reliable information for speech and voice research, as well as in medical diagnostics [1]. Segmenting the glottis is the first step in analyzing these records. The glottis, as the opening between the vocal cords in the larynx, is essential for phonation during speech production [2]. An accurate segmentation enables researchers and clinicians to analyze vocal fold dynamics, understand vocal function, and diagnose various voice disorders. In speech research, glottis segmentation allows for the investigation of vocal fold vibration patterns and fundamental frequency modulation, contributing to advancements in speech synthesis, voice recognition, and linguistic studies.

The detection of failed segmentations is of paramount importance in ensuring the reliability and trustworthiness of automated segmentation pipelines. Failed segmentations refer to instances where the algorithm fails to accurately delineate the desired anatomical structures or produces erroneous segmentations. These failures can arise due to myriad reasons, including image quality issues, algorithmic limitations, and inherent complexities in anatomical morphology. Since healthy glottal movements are often periodic, clinicians are especially interested in irregularities. Introducing them due to technical failure may impact the resulting features and lead to incorrect results during analysis. Any prospective clinical use case is a safety-critical scenario that bears the risk of harming the patient due to misdiagnosis.

Traditional approaches to address failed segmentations often rely on manual inspection and correction by medical experts or technical staff. However, manual intervention is labor-intensive, time-consuming, and prone to subjective biases. Consequently, there is growing interest in developing automated methods for the detection and localization of failed segmentations, leveraging machine learning techniques to enhance the efficiency and accuracy of segmentation quality assessment.

### Related Topics

- **Out-of-distribution detection** is a method used in machine learning to identify instances or data points that fall outside the training distribution of a model. It involves distinguishing data points that are within the model’s training data and those that are significantly different or anomalous. While it may be used for flagging instances that may cause erroneous predictions [3], it is also applied to detect anomalies [4].
- **Uncertainty estimation** in machine learning involves quantifying the uncertainty associated with model predictions by providing probabilistic estimates of confidence intervals or distributions rather than deterministic point predictions [5]. Techniques include Bayesian methods, dropout inference, ensemble methods, and Monte Carlo sampling, among others.

In both cases, the segmentation model would be modified or extended to return information about its behavior on the targeted task. The approach presented here, on the other hand, is to directly train a separate neural network to detect faulty segmentations. It therefore differs from uncertainty estimation and out-of-distribution detection, as it is meant as a corrective to the initial model. It is modular by being agnostic towards the neural network to which it is applied. Moreover, in contrast to out-of-distribution detection, the target is more specific towards a defined set of mistakes. This approach avoids the risk of rejecting segmentations that may be correct but differ from the latent distribution that the segmentation network learned.

Various similar approaches were proposed in Ref. [6] to train a model to detect when a segmentation is insufficient, as in the case of autonomous driving. Since their task was not binary, i.e., a case of semantic segmentation, they targeted the mean IoU of the different classes. They used an existing segmentation network with low accuracy to generate the training data and tried to predict the mean IoU. To solve imbalances in the distribution of the IoU, they employed a custom loss with a focus on outliers. Thereby, they achieved 85% accuracy in detecting failed segmentations of cityscapes using a single convolution neural network (CNN). Ref. [7] also tried to detect erroneous segmentations. Expert graded segmentations of spectral domain optical coherence tomography (SDOCT) images served as ground truth. Instead of predicting the IoU, authors trained a CNN (ResNet34) on the resulting classification problem. Their resulting model exhibited an accuracy of 92.4%. Ref. [8] trained a residual CNN to output the Dice score for cardiovascular magnetic resonance imaging segmentations. To generate erroneous segmentation data, they made use of random forests with different depths and an unsupervised method to approximate the accuracy with sparse label availability [9]. Authors reported a classification accuracy of 97% when they threshold the Dice score at 0.7 and 91% in the unsupervised setting. To our knowledge, there is no similar solution available yet for the application of glottis segmentation.

In this paper, a deep learning-based approach is presented that can detect bad glottis segmentations with an accuracy of 88.27% and return a quality score using a single classification network. A dataset with perturbed segmentations was created based on publicly available data for the purpose of using a new procedure and training a neural network using three custom losses. The following three contributions are presented in this paper:

- Knowledge-driven procedure for generating faulty glottis segmentations;
- Deep learning-based scoring and detection of faulty glottis segmentations;
- Comparison of adjustable custom losses for fine tuning.

Existing work is taken into account by going beyond the limitations of using a single model to generate data. Most importantly, the problem here can be regarded as an open-set problem [10]. Since the possible mistakes during segmentation are only defined negatively as deviations from the ground truth, they are potentially unbound. Using data that are sampled from a single source introduces a strong bias on this problem. This paper aims to improve beyond the limitations that may emerge when using a specific class of models on the available data. For this purpose, additional synthetic data are generated in a randomized and rule-based manner to prevent overfitting on specific types of errors. A more expressive custom loss is moreover incorporated to balance prospective bias. It is tailored towards a more comprehensive evaluation of the model’s accuracy. As in [6,8], an accuracy metric is predicted and subsequently turned into a classification problem using a threshold.

## 2. Materials and Methods

In this section, a comprehensive breakdown of the study’s methodology is provided. Initially, the focus is on the datasets utilized and their construction. This is followed by an explanation of the training process of the model, including the techniques and algorithms applied. Lastly, the selection and implementation of the loss function and metrics used for evaluating the model’s performance are presented.

**Figure 1:**
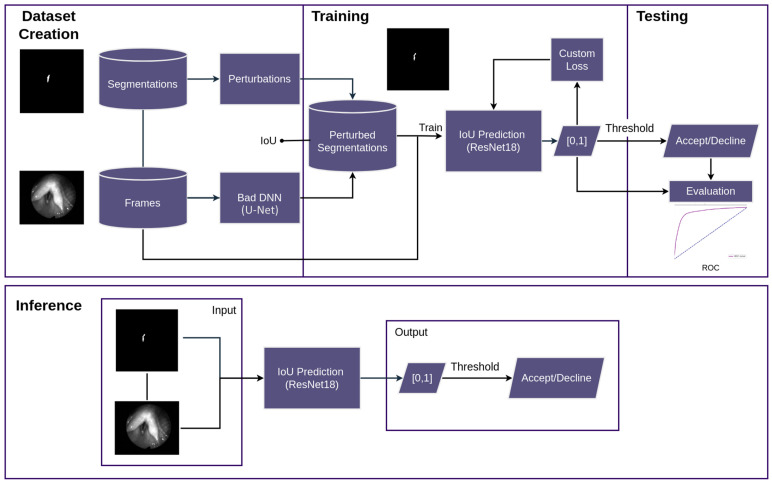
Basic overview of the method. First, bad segmentations were created using an insufficiently trained deep neural network and a newly proposed perturbation procedure. Second, a ResNet18 is trained to predict their IoU. At inference, segmentations are classified as good (“accept”) or bad (“decline”) based on an IoU threshold.

The approach presented here is depicted in Figure 1. First, the dataset is created. Then, training is performed. Subsequently, the results are evaluated. Lastly, in the case of inference only, the resulting neural network is used with an unseen frame and segmentation as the input. To create a dataset, three public datasets with manually created segmentations are used as a starting point. In order to obtain an appropriate dataset, a poorly performing deep neural network is used and a series of perturbations implemented using OpenCV [11] to perturb the good segmentations. These perturbations mimic mistakes that can occur when segmenting the glottis. The resulting dataset inhibits a mapping of IoU values to perturbed segmentations. During training, a ResNet neural network with a custom loss is trained to predict these IoU values using the original frame and the perturbed segmentation as the input. To test the resulting model, an evaluation is performed using the output score and a threshold to binarize the score. This binary value depicts the decision as to whether the segmentation should be accepted or declined as being too bad. During inference, these two outputs, i.e., score and decision, can be obtained from the resulting model.

### 2.1. Data

**Figure 2:**
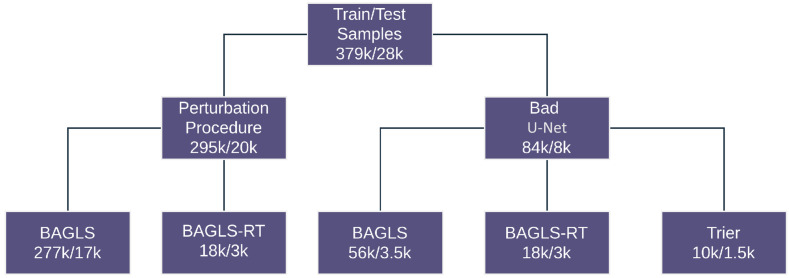
Distribution of the train/test data.

For training, three public datasets are used. Figure 2 depicts the data flow. The first dataset, from Trier University [12], consists of 130 unique videos with 100 images per video. It utilizes clinical data from the Department of Otorhinolaryngology and Head and Neck Surgery at the University of Munich and the Department of Otorhinolaryngology at Saarland University Hospital. Video recordings were captured using the HRES ENDOCAM 5562 from Richard Wolf GmbH, with a spatial resolution of 256 × 256 pixels and a frame rate of 4000 frames per second. The dataset includes 130 subjects, with 56 recordings from healthy individuals and 74 recordings from subjects with pathologies, further divided into organic and functional groups.

The second dataset used is the Benchmark for Automatic Glottis Segmentation (BAGLS) [13], which contains 59,250 randomly selected and manually segmented frames from 640 HSVs that were recorded in seven different locations in the USA and Europe. Their aim was to create a possibly diverse dataset that is representative of clinical cases. The creators included a diverse population in terms of age and gender, as well as healthy (380) and pathological (260) subjects. For the pathological cases, they included functional and organic dysphonia. Moreover, they used different cameras, resolutions, and light sources. Their dataset includes nasal as well as oral recordings with graded and varying quality.

A re-trained version of BAGLS was also included (BAGLS-RT [14]). BAGLS-RT was created to improve diversity in cases where specific groups were underrepresented in the original dataset. It comprises 267 HSV videos captured by eight distinct cameras and institutions, totaling 21,050 annotated images. It extends the original BAGLS dataset by incorporating five additional cameras, four novel light sources, a flexible endoscope, a unique frame rate, and 14 extra spatial resolutions. The dataset’s demographic breakdown includes a mean age of 42 with a standard deviation of 20 years, covering an age range from 18 to 93 years. It consists of 177 female and 90 male subjects, with 154 individuals exhibiting healthy voices and 123 individuals presenting various pathologies.

All three datasets offer more details in the connected papers and included metadata. Despite the necessity for further investigations, there is extensive research on the generalizability of these datasets, e.g., [14,15]. Following these, sufficient diversity can be assumed for representative results.

For preprocessing, the images are converted to grayscale, scaled to 256 × 256 pixels, and normalized between 0 and 1. The train/test split of the datasets is maintained as given in the original datasets. The perturbed segmentations were generated using these images. The training dataset consists of 379,000 frames in total and 28,000 for testing.

In order to turn the regression problem of predicting the IoU into a classification problem, a threshold value that identifies failed segmentations is needed. This value depends on purpose and application. For glottis segmentations, reference [12] generally considers an IoU of 0.7 as excellent. With reference to earlier work in the field, reference [15] argues that an IoU of 0.74 can be considered as sufficient for downstream tasks. Considering that human experts agreed with a mean IoU of 0.77 in a study conducted in [13], these values have to be considered as relatively strict. In addition, reference [16] stress the inter-observer variability of segmentations. Therefore, some margin for mistakes is considered, and segmentations below 0.6 are defined here to be failed.

**Figure 3:**
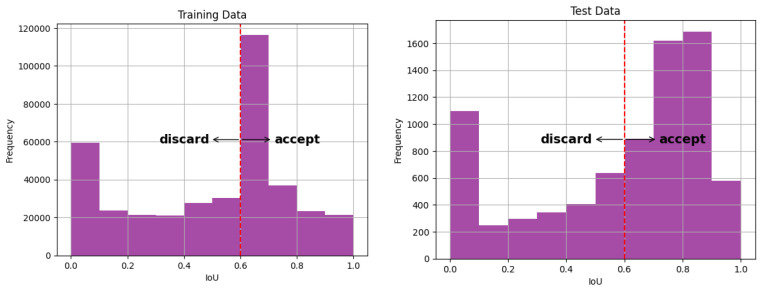
Overall score distribution of the train/test data. The train data are distributed around borderline cases using an insufficiently trained U-Net and a perturbation procedure. The test data are output by the U-Net.

Emphasis is given to borderline cases, with IoU close to 0.6 (Figure 3). The train data have a mean IoU score of 0.5 with a standard deviation (std) of 0.284. For the test data, a setting with a mean IoU of 0.55 and a std of 0.3 is used, as well as a uniformly distributed setting.

### 2.2. Generating Bad Segmentations Using a Neural Network

To induce errors in the segmentation outputs of a standard U-Net model [17] trained on the Trier dataset, it was subjected to extensive data augmentations, intentionally degrading its performance under challenging conditions. Augmentations involved rotations (x: −10 to 10°, y/z: −5 to 5°), gamma correction (range: 80 to 120), brightness/contrast changes (brightness limit: ±20%, contrast limit: ±20%), and Gaussian noise (variance limit: 0.10 to 0.50). Gradually increasing the augmentation intensity using a factor amp, sufficiently low accuracy was achieved by setting the amp to 7 for perturbed segmentations. This methodology, informed by a prior study [18], allowed us to explore the model’s response to various noise and distortion sources, simulating real-world conditions. Upon evaluating the trained model on BAGLS, BAGLS-RT, and the Trier dataset, deviations from ground truth annotations were observed, generating diverse erroneous segmentations for further analysis. The resulting training dataset, augmented from the Trier dataset, exhibited a mean IoU of 0.55 with a standard deviation of 0.3, while the test dataset, comprising samples from multiple datasets, displayed a mean IoU of 0.58 with a standard deviation of 0.3. The diversity of error source due to different data augmentations should result in a more diverse set of errors occurring during inference, thereby generating a suitable dataset to learn to detect faulty segmentations.

### 2.3. Generating Bad Segmentations Using Perturbations

**Figure 4:**
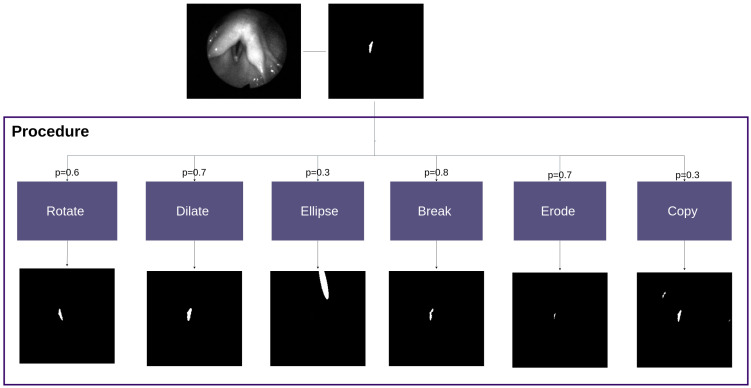
Scheme for the procedure to perturb the ground truth. Six different perturbations are applied with a probability of p.

Since sampling from only one neural network results in suboptimal generalizability, additional perturbations are implemented using OpenCV. Also, a goal is also to achieve non-obvious segmentation errors, which are not detectable without the original frame. The procedure consists of 6 types of perturbations that are applied randomly with probability p. Figure 4 shows the whole procedure. For details, see Appendix A.

- **Rotation** is performed on the initial segmentation to maintain the shape features but create implausibility in terms of its position.
- **Dilatation** simulates over-segmenting and smoothed contours.
- **Erosion** simulates under-segmentation and irregular contours.
- **Breaking** the segmentation simulates holes in the segmentation and random missing fragments.
- **Copying** the segmentation forces the network yet again to focus on the position. The copied segmentation is also resized randomly.
- Inserting an **ellipse** into the segmentation is used to introduce a shape that looks similar to the glottis. It is applied more often in cases where there is nothing segmented in the ground truth, to simulate incorrect segmentations when the lips are closed.

The range [0,1] is split into 6 bins with different probabilities, therefore resulting in a suitable distribution of scores (see Figure 3). The target score is then drawn for each segmentation. Sampling is performed from these perturbations by iterating and picking them randomly until the target score is achieved. The code for the procedure is available online (https://github.com/ADadras/GlottisSegPerturbation, not yet online, will be provided, accessed on 25 April 2024).

### 2.4. Training

As architecture, a plain ResNet18 [19] was used. The ResNet architecture allows us to build neural networks with more depth because it solves the problem of vanishing gradients by introducing residual connections. Due to increasing abstraction in later layers and the limited receptive field of convolution operations, important information from the input image may be lost. Residual connections establish an information flow from earlier layers to deeper layers. This forces the network to perform residual learning, i.e., learn the relationship of the current layer to the earlier subnetwork. Particularly in biomedical imaging, where global context may be important, this has been shown to be effective [20].

A single frame and segmentation were used as the input to the neural network, thereby resulting in two channels as input (grayscale frame and segmentation) instead of RGB channels as in the vanilla ResNet. The softmax on the output was removed. Instead, the fully connected layer is reduced to one output value. The resulting architecture is outlined in Table 1.

The problem is treated the during training phase like a regression problem, with the IoU as the target value, and a threshold function is added for binary classification during evaluation.

#### Loss

To incorporate the desired acceptance rate, two intuitions are incorporated into the loss function:

1. Incorrectly classifying the segmentation below or above the acceptance rate should have a bigger penalty. Detecting bad segmentations is of greater interest than detecting the accurate score.
2. Accurately predicting the score below the acceptance rate is more important than the above. It is not as important how good a segmentation is as how bad it is, especially when it is detected as failed. This intuition allows for potentially automatic error correction in the future.

**Figure 5:**
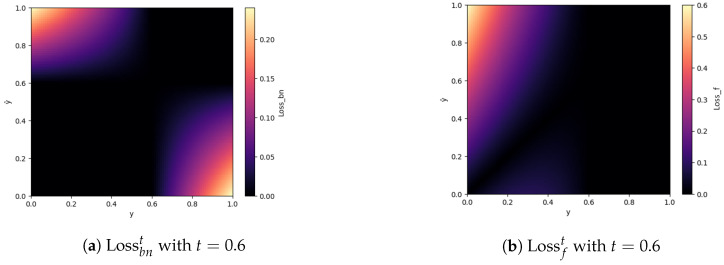
For training, two terms were used to concentrate on two aspects. As visible from the heat map, the term in (**b**) puts a bigger emphasis on cases below the acceptance rate of *t* = 0.6. On the other hand, (**a**) is making the network concentrate on cases that are accepted or declined incorrectly, i.e., wrongly above or below *t*. The black areas are zero and are when the prediction is correctly identified as above or below the acceptance rate *t* = 0.6).

To address intuition (1), a loss term was designed to impose a stronger penalty when the prediction is incorrectly classified as above or below the threshold. This functionality is depicted in Figure 5a. The loss function remains zero when the classification as accepted or declined is correct. Only for false classification does it becomes active. The borders of the acceptance threshold are denoted by *t* in the equation. The point at which this aspect of the loss becomes active is effectively defined, i.e., greater than 0.

(1) $${\mathrm{Loss}}_{bn}^{t}\left(y,\hat{y}\right)=\frac{1}{N}\sum_{i=1}^{N}(\max\left(0,{\hat{y}}_{i}-t\right)\;\cdot \;\max\left(0,t-y_{i}\right)+\max\left(0,t-{\hat{y}}_{i}\right)\;\cdot \;\max\left(0,y_{i}-t\right))$$

In a similar vein, intuition (2) is integrated into the approach by introducing mean absolute error (MAE) that is activated when the target value falls below 0.6, as illustrated in Figure 5b. This functionality is accomplished by setting the function to 0 if the target *y* is bigger than *t*, thereby using only the part that affects the cases below it. The loss should be higher for deviations from the ground truth occurring below the acceptance threshold *t*.

(2) $${\mathrm{Loss}}_{f}^{t}\left(y,\hat{y}\right)=\frac{1}{N}\sum_{i=1}^{N}(\max\left(0,(t-y)\right))\;\cdot \;\mathrm{MAE}\left(\hat{y},y\right)$$

The loss terms are combined with an MSE (mean squared error) loss, and two weight factors, $\lambda_{0}$ and $\lambda_{1}$, are added. The first one adjusts the ratio of the MSE to the custom loss term. The second adjusts the ratio of the two components of the custom loss term.

(3) $$\mathrm{Loss}\left(y,\hat{y}\right)=\lambda_{0}\;\cdot \;\mathrm{MSE}\left(y,\hat{y}\right)+\left(1-\lambda_{0}\right)\;\cdot \;\left(\lambda_{1}\;\cdot \;{\mathrm{Loss}}_{bn}^{t}\left(y,\hat{y}\right)+\left(1-\lambda_{1}\right)\;\cdot \;{\mathrm{Loss}}_{f}^{t}\left(y,\hat{y}\right)\right)$$

### 2.5. Metrics

As a measure to evaluate the results, the MSE of the predicted IoU and the IoU of the target segmentation in regards to the ground truth segmentation were used. The IoU is a measure of segmentation overlap, using the true positive (TP), false positive (FP), and false negative (FN) number of pixels.

$$\mathrm{IoU}=\frac{\mathrm{Area}\;\mathrm{of}\;\mathrm{Intersection}}{\mathrm{Area}\;\mathrm{of}\;\mathrm{Union}}=\frac{TP}{TP+FP+FN}$$

The network is also evaluated with respect to its ability to distinguish between the two classes by employing a threshold that can be seen as a hyperparameter. It can be adjusted depending on the desired position on the receiver operating characteristic (ROC) curve. To evaluate the resulting classification, the IoU derived from the literature was used. Therefore, the IoU is discretized, with 0.6 or higher as accepted and below as rejected.

In a safety-critical [21] use case, such as in the clinical setting, sensitivity and specificity are of special importance. False negatives or positives may impact the user experience differently and potentially endanger individuals. A redundant check for a broken frame might be neglectable if missing broken frames lead to faulty results and an incorrect diagnosis. In such a case, higher sensitivity is desirable. Moreover, the downstream task may require different sensitivity/specificity. Only filtering out good cases (e.g., [22]) with a high certainty can reduce workload, and this requires high specificity.

## 3. Experiments

Augmentation techniques such as gamma correction, brightness/contrast adjustment, Gaussian noise addition, rotation, and flipping were employed using the Albumentations library [23] to enhance dataset diversity and prevent overfitting. This is of particular importance because the same frame was used multiple times in the dataset.

The ResNet with 18 layers was selected. Experiments showed no improvement or worse accuracy using more parameters or different architectures. The smaller model requires less training time and inference and is therefore preferable.

The training specifications are summarized in Table 2. The learning rate was set to 0.0001. Higher learning rates, such as 0.001, showed instabilities, where the ResNet would collapse to a local minimum. The training was conducted for 100 epochs to search the parameter space thoroughly. The training loss showed a steady decrease in later epochs as well, meaning that network parameters were still updated. A batch size of 18 was used as a compromise between regularization and memory usage. Model initialization was performed using the Kaiming method [24]. Weight initialization in general improves convergence properties. The Kaiming method, moreover, is especially effective with rectified linear units (ReLUs) as the activation functions, as they are used in the ResNet. The Adam optimizer was utilized, with beta1 and beta2 values set to the most commonly used values of 0.9 and 0.999, respectively. For training, a cyclic learning rate scheduler was used to optimize convergence. It has shown to be an effective choice for segmentation tasks of the glottis [13,18]. Gradient clipping was set to 1 to ensure no exploding gradients.

The model is written in PyTorch and trained on an A100 graphics processing unit (GPU; NVIDIA, Santa Clara, CA, USA). Early stopping is applied after 20 epochs if the last best model is more than 10 epochs ago. During the testing phase, the best model was picked using the MSE.

Training is conducted using various loss settings to evaluate the influence of different terms. Table 3 outlines the different settings. Given that the data are centered around 0.6 and thresholding for binary accuracy is performed at this value, the threshold *t* is maintained at 0.6 for these experiments.

The effect of the *t* parameter on the loss is tested separately. As a baseline, the balanced λ parameters are fixed. The parameter *t* is varied slightly around the threshold of 0.6 to see how strongly it affects the result. Table 4 shows the choice of parameters.

The model is fine-tuned according to the intentions that were incorporated in the loss. Therefore, both the results for the absolute accuracy and the behavior in different data segments are analyzed.

### Evaluation

All models are evaluated on the data obtained with the U-Net. To show the effect of the perturbation procedure, two additional settings on the MSE baseline are compared separately.

- Procedure only: only data obtained from the procedure (see Figure 2).
- Stratified with procedure: the test data are stratified by using additional data from the procedure to have a uniform setting.

Three neural networks trained on the three data origins (U-Net, procedure, and both) are compared.

## 4. Results

Results indicate that incorporating the custom loss functions alongside the mean squared error loss leads to notable improvements. Notably, employing a weakly weighted MSE combined with solely the custom loss function ${\mathrm{Loss}}_{f}$ achieves an accuracy of 88.27% with an MSE of 0.0274, representing a significant enhancement over plain MSE. While the MSE appears to stabilize accuracy, the additional loss terms contribute to improved specificity and sensitivity, even when combined with MSE loss.

Furthermore, the effect of the threshold *t* during model training on performance shows minor fluctuations in performance within a close range of t=0.6. A visible drop in MSE is observable when *t* deviates significantly from this value. This highlights the importance of maintaining *t* close to the desired threshold for optimal prediction accuracy.

Additionally, the effect of the perturbation procedure on model performance is investigated. Evaluation on data solely from the U-Net or solely from the procedure demonstrates moderate accuracy, while combining both sources results in a notable improvement. The use of perturbed data during training leads to significant enhancements in accuracy and MSE, underscoring the positive impact of the procedure on model performance.

Finally, the performance characteristics of the model are examined, particularly in challenging cases where segmentations are on the edge between acceptance and rejection. Results indicate that the choice of loss function plays a crucial role, with the model focusing on correcting particularly challenging segmentations.

### 4.1. Effect of Using Different Losses

Table 5 shows the general accuracy and MSE for different loss settings. Incorporating custom loss functions alongside MSE loss yields notable improvements in model performance. The usage of a weakly weighted MSE and only ${\mathrm{Loss}}_{f}$ achieves an accuracy of 88.27%, with an MSE of 0.0274. In comparison to the plain MSE loss, it achieves an improvement in accuracy of approximately 2%. Using only the custom loss terms decreases accuracy by 2% but shows significantly better specificity and sensitivity. While the MSE seems to have a stabilizing effect on the accuracy, the additional loss terms improve specificity and sensitivity. Moreover, they improve the MSE when combined with the MSE loss.

### 4.2. Effect of *t* during Training

Table 6 presents the results of experiments conducted to evaluate the effect of varying threshold *t* on model performance. We observed minor fluctuations in performance when varying *t* within a close range of 0.6, but MSE dropped visibly when *t* deviated too far from this value. This suggests that maintaining *t* close to the desired threshold (in this case, 0.6) is crucial for achieving optimal prediction accuracy.

### 4.3. Effect of Perturbation Procedure

First, the accuracy is investigated when the networks are only evaluated on the test data from the U-Net (Table 7). Using only the procedure does still perform decently, with almost 80% accuracy, though the MSE shows a bigger gap for predicting the accurate score. The U-Net, which has been trained on the same data source as it is being evaluated on, performs roughly 4% better, and the combination of both improves yet again by 2%. The usage of the procedure does have a positive impact on the performance.

Next, the usage of segmentations obtained from the perturbation procedure in the stratified setting is compared. A significant improvement in accuracy and MSE is observed when the data from the procedure for training are also included. As shown in Table 8, accuracy increased by roughly 11%, while MSE decreased by a factor of 3 when using perturbed data compared to no perturbed data. The improvement comes as no surprise, but the large gap of 11% is notable.

### 4.4. Performance Characteristics

**Figure 6:**
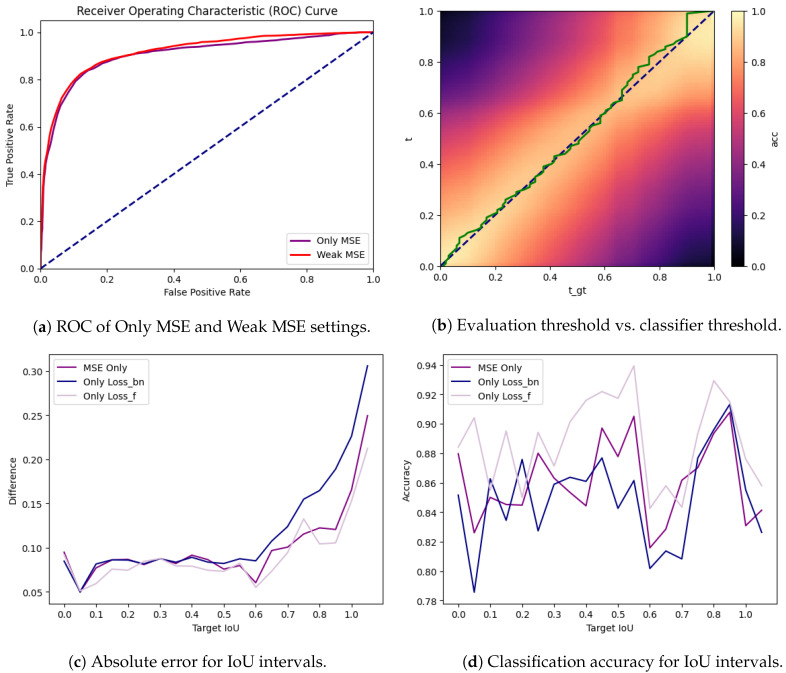
Subfigure (**a**) depicts the receiver operating characteristic (ROC) curve of the model. It shows the relationship between sensitivity and specificity of the classifier. Subfigure (**b**) shows the accuracy of the classifier depending on the threshold ($t_{gt}$) on which the continuous IoU of the ground truth and the output of the model is binarized using the threshold *t*. The green line shows the maximum accuracy, depending on the chosen threshold during inference. Subfigure (**c**) shows the mean difference between output and ground truth for different IoU intervals of the ground truth. Subfigure (**d**) shows the accuracy of the model for different IoU intervals of the ground truth.

As expected, the performance drops in hard cases where the segmentation is on the edge between acceptance (>0.6) and rejection (<0.6). In Figure 6a, it is possible to check if the binary accuracy of the model can be improved by choosing a threshold for the model during inference that differs from the desired threshold. As shown in Figure 6b, they align almost all of the time, although there is room to increase the true positive rate by accepting a higher false positive rate.

The model performs better or worse depending on how close the segmentation in question is to the defined threshold of 0.6. As visible in Figure 6c,d, the model focuses on getting particularly bad segmentations correct. The choice of the loss therefore plays an important role. As visible in Figure 6c, the model pays less attention to the accurate score of the segmentations when only ${\mathrm{Loss}}_{bn}$ is applied. The mean absolute error rises around the cases with very high IoU. The model can apparently separate them without having to learn too much about their finer details. On the contrary, the usage of ${\mathrm{Loss}}_{f}$ causes the model to pay more attention to getting the score in the lower intervals right. Interestingly, this also causes it to perform on par with or even better than the pure MSE in higher IoU intervals. In Figure 6d, one can see accordingly the expected behavior when looking at classification accuracy. Using only the ${\mathrm{Loss}}_{bn}$ is insufficient to get the finer details. ${\mathrm{Loss}}_{f}$ seems to succeed in most cases, but a combination of both can be viable, as shown above.

**Figure 7:**
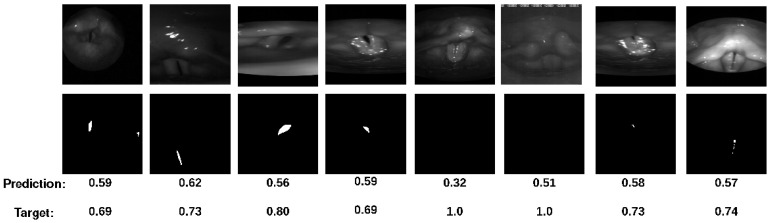
Examples of cases where the model trained on the MSE fails but the balanced network classifies them correctly.

Cases with small segmentations are especially hard for the network to assess because small changes have a large impact on the IoU. Figure 7 shows cases the MSE network fails to classify correctly, but most of the networks trained with additional loss terms perform better. The additional loss terms generally seem to improve the model in these cases.

#### Inference Time

For glottis analysis, videos of up to a couple of seconds are the most relevant. HSV frame rates can go up to 10,000. With a moderate frame rate of 4000 per second and 3 s of recording, that would be 12,000 frames. For evaluation, the model is run on a consumer GPU (NVIDIA GTX 2080) with 8 GB of VRAM (NVIDIA, Santa Clara, CA, USA). The trained ResNet can process a minimum of 400 frames per second with this setting. Because of the novelty of the presented approach, the only alternative is checking the data manually. In comparison, a human reader’s visual system can process up to 12 frames per second reasonably if it wants to catch errors [25]. Higher frame-rates lead to overlooking intermediate frames as motion. That is a ratio of 400 to 12, which makes the ResNet approximately 33 times faster. For a video of 12,000 frames, the ResNet would therefore need 30 s, whereas the human reader would need 1000 s (or 16.6 min). Considering that the human reader has to pause the video to note broken frames and that it is possible to accelerate the ResNet by using multiple instances in parallel or a faster GPU, this difference increases even more.

## 5. Discussion

In general, the presented approach improved over the pure MSE with the custom loss by 2% accuracy. Tweaking results using the loss parameters brought minor improvements in terms of accuracy but significantly different specificity and sensitivity. The fact that the MSE is lower using a combination of the losses instead of directly training on the MSE indicates that the network learns information that it would probably have overlooked otherwise.

The model achieves an accuracy of 88.27% on the dataset, which decreases for the most challenging cases, falling between IoU values around the threshold of 0.6. It is important to interpret this result in light of the inherent difficulty in classifying cases near the threshold, which tend to be ambiguous. Given that the dataset is centered around these values, it is evident that the task becomes more challenging as a result. During evaluation with less biased data, it is possible to see the model perform within a reasonable accuracy. The prediction of the IoU differs on average for cases with low IoU, around 0.1, and high specificity.

The intuition is to concentrate on predicting a more accurate IoU for failed segmentations. This has been shown to be effective in the performance characterization and opens up applications that assess and improve on failed segmentations without human intervention.

The inter-observer variability that poses a natural noise factor in the application is especially severe in cases where it is questionable whether there is a glottis visible at all. This can be the case when the glottis is either closed or nearly closed and when the image quality is insufficient. The model here does not show a visible performance drop for the case of low lighting and performs better on the cases with a small glottis area using the custom loss. Rather, its performance drops in corner cases where the segmentation errors are non-obvious, i.e., look plausible despite very low IoU. Nonetheless, there is still room for improvement.

The procedure avoids introducing unseen frames into the dataset, thereby attributing the enhanced performance of the network to the perturbed segmentations. This approach refrains from providing the network with novel information about the glottis itself. Consequently, it mitigates the risk of the network memorizing a frame-to-score mapping or a specific pattern from the neural network, ensuring a more robust learning process. By randomizing the distribution of perturbations, a challenging learning task is imposed on the network, compelling it to capture the desired relationship between intersection over union, glottis, and segmentation.

The choice of *t* had only minor effects on the result. We attribute this to the MSE loss, which is probably being favored during optimization. Also, the dataset has a strong bias towards the desired threshold. In cases where the training distribution of the data might be different from the target distribution and more weight is put on the terms affected by *t*, the effect of the parameter is expected to be higher.

As an alternative to ResNet18, training was also performed using ResNet32, ConvNext, and SwinTransformer. These either did not converge or did not show any advantage. Seeing no advantage in adding redundant parameters, the decision was made to stick with the simpler model.

Existing segmentation models perform well on the presented datasets but fail to generalize to a degree that would make manual observation obsolete. The baseline presented in the BAGLS paper [13] is based on a standard U-Net CNN, as it is the most commonly used network architecture for biomedical images. It already shows a mean IoU of 0.83. Nonetheless, the need for the follow-up BAGLS-RT paper showed the necessity of expanding the original dataset and the ability to retrain models. Ref. [26] argues that, rather than fixed models, the ability to adapt to ever-changing environments is key to the success of AI application. Demographics, devices, etc., do not stay the same. In the same manner, Ref. [15] observes the long-term performance of their network for glottis segmentation in a clinical setting. They successfully apply a scheme for continual training, thereby reducing artifacts by up to 81%. Detecting failed cases automatically, as presented here, can assist with improving existing systems. The agnostic nature of the model here allows for maintaining it even after modifying the segmentation model.

While the perturbation procedure works fine for the detection problem, it is far from complete. It is questionable if the quality of a segmentation is accurately measured by a simple quantitative metric such as IoU. Ref. [27] shows how every quantitative metric has different strengths and weaknesses. IoU primarily measures the overlap between individual pixels rather than considering the overall structure of segments. Different types of mistakes make have a bigger impact on the resulting features, despite showing the same IoU. In the case of pixel-wise metrics, they show correct measurements for segmentations on a global level, yet they overlook region-based agreement and boundary accuracy. Consequently, they struggle to distinguish models that enhance region boundary alignment with ground truth. In the case of the PVG, for example, where the distance to the midline is of importance, incorrectly segmented areas in distant areas have a bigger impact than under/over-segmentation. The IoU ignores the shape and internal structure of objects, focusing exclusively on the overlap area. Therefore, shape features, referring to the outline of the glottis segmentation, may not be accurately addressed. Ref. [13] also points out that the size of the segmentation heavily influences the IoU. In cases where the glottis is almost not visible because it is almost closed, only a few pixels are segmented. Small disagreements can result in a very low score.

Future research would need to go beyond pure IoU prediction. For this purpose, quantitative studies are necessary regarding the relationship between IoU and the most commonly performed downstream tasks. While it is difficult to specify these due to the lack of standardized procedures for glottis analysis, there are promising approaches. In [28], the most important features for classification are outlined. These can serve as a reference to find out about the correlation between IoU and prospective features. More expressive metrics than the IoU [29,30] could enhance the result. In [31], a general approach is described to overcome the weaknesses of the IoU and adjust the metric to the given task. Another possible direction could be learning the metric [32] using human graders. While this may be labor-intensive, it would offer a tailored evaluation metric. Another possible research direction could be the usage of different algorithms. The study presented here was limited to deep learning and standard architectures. Rule-based methods and more sophisticated architectures could also be viable. Lastly, the detection of the specific error regions could be valuable follow-up research. In [33,34], a generative model is applied to reproduce the original image from the segmentation. Error regions are then identified, where this reproduced image differs from the original image significantly.

## 6. Conclusions

Overall, this paper presents the first fully automated method for evaluating glottis segmentation quality and identifying instances of failure. The experiments show the feasibility of detecting failed glottis segmentations using neural networks and synthetic data. Additionally, a comprehensive and openly accessible procedure for generating a suitable dataset is provided, demonstrating the efficacy of both dataset construction and loss design. Adjusting the loss and the distribution of training data resulted in different performance characterizations that can be tailored to the use case. More research on the expressiveness of the IoU for the task of glottis analysis is necessary to determine which metric is appropriate.

## Figures and Tables

**Table 1:** Specifications of the modified ResNet18. Two instead of three input channels were used for the grayscale segmentation and frame. Furthermore, only one output node is used for the score.

Layer	Output Size/Operation
Input	2×256×256 image
Conv1	64×112×112, 7×7 conv, stride 2
Maxpool1	64×56×56, 3×3 maxpool, stride 2
Layer1	64×56×56 residual block (2 layers)
Layer2	128×28×28 residual block (2 layers)
Layer3	256×14×14 residual block (2 layers)
Layer4	512×7×7 residual block (2 layers)
Avgpool	512×1×1, global average pooling
Fc	(512,1) fully connected layer

**Table 2:** Training specifications for ResNet18.

**Optimizer**	Adam
**Initialization**	Kaiming
**Gradient Clipping**	1
**Scheduler**	Cyclic
**Learning Rate**	0.0001
**Batch Size**	18
**Data Augmentations**	Gamma, Brightness/Contrast, Gaussian Noise, Rotation

**Table 3:** Experimental settings with different loss term weights.

Training Setting	$\lambda_{0}$	$\lambda_{1}$
Only MSE	1	0
Only ${\mathrm{Loss}}_{f}$	0	1
Weak MSE, Only ${\mathrm{Loss}}_{f}$	0.25	1
MSE, Only ${\mathrm{Loss}}_{f}$	0.5	1
Only ${\mathrm{Loss}}_{bn}$	0.0	0
Weak MSE	0.25	0.5
Weak MSE, Only ${\mathrm{Loss}}_{bn}$	0.25	0
MSE, Only ${\mathrm{Loss}}_{bn}$	0.5	0
Balanced	0.5	0.5
No MSE	0	0.5

**Table 4:** Experimental settings with different *t* parameters.

*t*	$\lambda_{0}$	$\lambda_{1}$
0.3	0.5	0.5
0.5	0.5	0.5
0.55	0.5	0.5
0.6	0.5	0.5
0.65	0.5	0.5
0.7	0.5	0.5
0.8	0.5	0.5

**Table 5:** Results for different loss settings sorted by accuracy (higher is better).

Training Setting	MSE	Accuracy	Specificity	Sensitivity
Weak MSE, Only ${\mathrm{Loss}}_{f}$	0.0274	**88.27%**	91.53%	64.08%
Weak MSE	0.0296	87.44	87.21%	62.53%
Weak MSE, Only ${\mathrm{Loss}}_{bn}$	0.0281	87.01%	91.56%	65.93%
MSE, Only ${\mathrm{Loss}}_{bn}$	0.0304	86.87%	92.71%	67.71%
Only ${\mathrm{Loss}}_{bn}$	0.0307	86.84%	92.71%	67.71%
MSE, Only ${\mathrm{Loss}}_{f}$	**0.0267**	86.77%	88.06%	62.03%
Balanced	0.0282	86.71%	91.15%	65.04%
Only MSE	0.0293	86.07%	86.26%	63.35%
No MSE	0.0324	84.88%	**95.08%**	**70.31%**
Only ${\mathrm{Loss}}_{f}$	0.0342	84.97%	75.47%	55.22%

**Table 6:** Results of using different *t* parameters during training on the balanced setting ($\lambda_{0}=0.5$, $\lambda_{1}=0.5$, t=0.6).

*t*	MSE	Accuracy
0.3	0.0336	86.47%
0.5	0.0314	85.88%
0.55	0.0316	86.54%
0.6	0.0282	86.71%
0.65	0.0316	86.57%
0.7	0.0322	85.94%
0.8	0.0304	86.40%

**Table 7:** Evaluation of only data from U-Net and trained without data from the procedure using only MSE loss.

Training Setting	MSE	Accuracy
Procedure and U-Net	**0.0293**	**86.07%**
Only U-Net	0.0372	83.9%
Only Procedure	0.0691	79.6%

**Table 8:** Evaluation of data from both sources and trained without data from the procedure using only MSE loss.

Training Setting	MSE	Accuracy
Procedure and U-Net	**0.0248**	**86.66%**
Only U-Net	0.0877	67.22%
Only Procedure	0.0357	83.89%

## Data Availability

All datasets used are, at the time of the experiments, publicly available.

## Appendix A. Perturbation Algorithms

To generate the perturbed segmentations in Algorithm A1, the functions are iterated, which change the segmentation and are applied randomly with random parameters within a defined range. If the segmentation has less than 20 segmented pixels, the ellipse is inserted with a probability of 30%. A range of IoU is chosen in Algorithm A2 and iterated until the segmentation is in the desired range. This is done for a maximum of 10,000 iterations and repeated 5 times if a fitting segmentation cannot be found. If so, it simply stays with the current perturbation.

**Algorithm A1:** Perturb Segmentation
**Data:** seg
**Result:** Perturbed seg

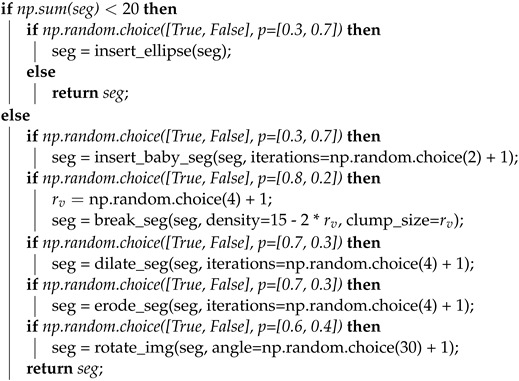

**Algorithm A2:** Perturb Evenly
**Input:** seg, meta_iter = 0
**Output:** seg_new
IoU_Classes = [0, 0.4, 0.6, 0.65, 0.7, 0.75, 1];
IoU_min = pick with p = [0.2, 0.5, 0.2, 0.1, 0, 0] from len(IoU_Classes);
IoU_Range = IoU_Classes[IoU_min, IoU_min + 1];
score = −1;
max_iter = 10,000;
